# Investigation of rare variants in *LRP1, KPNA1, ALS2CL *and *ZNF480* genes in schizophrenia patients reflects genetic heterogeneity of the disease

**DOI:** 10.1186/1744-9081-9-9

**Published:** 2013-02-20

**Authors:** Loubna Jouan, Simon L Girard, Sylvia Dobrzeniecka, Amirthagowri Ambalavanan, Marie-Odile Krebs, Ridha Joober, Julie Gauthier, Patrick A Dion, Guy A Rouleau

**Affiliations:** 1 Center of Excellence in Neuroscience of Université de Montréal and the Department of Medicine, Montreal, Quebec, Canada; 2 Centre de Recherche du Centre Hospitalier de l'Université de Montréal (CRCHUM), Montreal, Quebec, Canada; 3 Université de Montréal, Faculty of Medicine, Department of Pathology and Cell Biology, H3C 3J7, Montreal, Quebec, Canada; 4 Research Center, CHU Sainte-Justine, H3T 1C5, Montreal, Quebec, Canada; 5 INSERM Laboratory of Pathophysiology of Psychiatric Diseases, Center of Psychiatry and Neurosciences, U894, Sainte-Anne Hospital, Paris, France; 6 University Paris Descartes, Faculty of Medecine Paris Descartes, Centre d’évaluation et de recherche clinique, Sainte-Anne Hospital, Paris, France; 7 Douglas Mental Health University Institute, Department of Psychiatry, McGill University, Pavilion Frank B. Common, Verdun, Montreal, Quebec, Canada

**Keywords:** Schizophrenia, *De novo* mutation, *LRP1*, *ALS2CL*, *KPNA1*, *ZNF480*

## Abstract

**Background:**

Schizophrenia is a severe psychiatric disease characterized by a high heritability and a complex genetic architecture. Recent reports based on exome sequencing analyses have highlighted a significant increase of potentially deleterious *de novo* mutations in different genes in individuals with schizophrenia.

**Findings:**

This report presents the mutation screening results of four candidate genes for which such *de novo* mutations were previously reported (*LRP1*, *KPNA1*, *ALS2CL* and *ZNF480*). We have not identified any excess of rare variants in the additional SCZ cases we have screened.

**Conclusions:**

This supports the notion that *de novo* mutations in these four genes are extremely rare in schizophrenia and further highlights the high degree of genetic heterogeneity of this disease.

## 

Schizophrenia (SCZ) is a neurodevelopmental psychiatric disorder that is characterized by severely impaired cognitive processes causing hallucinations, delusions and altered emotional reactivity that disturb social behavior. This disorder is highly prevalent and according to the National Institute of Mental Health affects 1.1% of the U.S. adult population. The genetic factors predisposing to SCZ have not been fully elucidated but twin, adoptee and family studies jointly suggest that genetics is important with a heritability estimated to be up to 80% [1,2]. Multiple approaches have been used to identify common and rare SCZ-predisposing variants using candidate gene and whole genome-scale studies [3,4]. However neither large-scale sequencing projects, looking for rare penetrant variants, nor genome wide association studies, looking for common variants, have accounted for a significant fraction of the heritability of SCZ. In light of this limited success, it was hypothesized that deleterious *de novo* mutations in any of several different genes could explain the high global incidence of SCZ despite a reduced reproductive fitness. Our group first reported the presence of a significant excess of potentially deleterious *de novo* mutations in 401 synaptic genes using Sanger sequencing in a cohort of SCZ and autism patients [5]. We have later confirmed an excess of exonic *de novo* mutations and more particularly of nonsense variants in 15 SCZ trios (probands and parents) using exome sequencing. Interestingly, 4:11 ratio of nonsense to missense mutations is significantly higher than the expected 1:20 ratio (P = 0.005467), and according to the Human Gene Mutation Database, the expected ratio of nonsense to missense among all mutations reported to cause Mendelian diseases is 1:4 which in line with what we found (P > 0.05, not significant). This identified four candidate genes (*LRP1*, *KPNA1*, *ALS2CL* and *ZNF480*) possibly involved in SCZ [6]. The present report describes the mutation screening of these four genes in additional 475 SCZ cases and 189 controls.

## 

Probands were individually interviewed and their diagnosis was based on the Diagnostic and Statistical Manual of Mental Disorders, Fourth Edition. Exclusion criteria included patients with psychotic symptoms caused by alcohol, drug abuse or other clinical diagnosis. All samples were collected through informed consent following the approval of their respective institutional ethics review committees. An initial cohort of 189 SCZ patients of European Caucasian ancestry was used in this study. An additional cohort of 285 SCZ subjects, also of European Caucasian ancestry, was used for the screening of *ALS2CL* and *ZNF480* genes. All coding regions and splice site junctions of *LRP1*, *KPNA1*, *ALS2CL* and *ZNF480* genes were amplified and then sequenced using Sanger sequencing technology. Variant detection analysis was done using Mutation Surveyor (v. 3.23, Softgenetics) and rare exonic variants were confirmed by re-amplification of the relevant fragment in both probands and parents (when available) using forward and reverse oligos.

## 

The first gene, *LRP1* (NM002332, 89 exons) encodes for *Homo Sapiens* low density lipoprotein receptor-related protein 1 and is located at 12q13-14. We identified four rare *LRP1* variants (S278I, R379H, M1795I and G3948D) absent from public databases (dbSNP [7], 1000 genome project [8] and Exome Variant Server (EVS) [9]) and two additional variants (G169D and A2160T) only reported in EVS at a very low frequency (Table 1). Interestingly, the missense G169D is predicted to affect protein function by SIFT (0.01) and Polyphen (2.337) predicting softwares [10,11]. Unfortunately the parents’ DNAs were not available for an inheritance study. We also identified eight other *LRP1* variants that were previously deposited in EVS database and their minor-allele frequencies ranged from 1 to 159 / 10 755 to 10 599 total alleles. Two of these, G3725E and R1993W, are predicted to affect protein function despite minor-allele frequencies of 12/ 10 746 and 25/ 10 733, respectively. However, the fact that G3725E is transmitted by an unaffected mother, suggests it is unlikely to play a major role in SCZ. Unlike the original published findings, we did not find any highly damaging variant (ie. nonsense or insertion/deletion) in *LRP1* gene. The *KPNA1* gene screening (NM002264; 14 exons), which encodes for karyopherin alpha 1 protein and is located at 3q21, did not lead to the identification of rare variants in our SCZ cohort. The sequencing of *ALS2CL* (NM147129; 26 exons), encoding for ALS2 C-terminal like protein and located at 3p21.31, revealed one nonsense mutation (E65X) in one SCZ patient. Because of this *ALS2CL* variant, we opted to screen the full gene in 286 additional SCZ patients and 189 control individuals. We observed a minor-allele frequency of 4/475 SCZ patients and of 1/189 controls for E65X. This variant has been lately added to dbSNP (rs139496961) and EVS databases with a minor allele-frequency of 20/10 738. The presence of E65X in a control individual from our ethnically-matched control cohort makes *ALS2CL* gene less likely to predispose to SCZ. We also observed three additional rare *ALS2CL* variants in our 189 initial cases (T268S, T460M and P580S) but their pathogenicity is unlikely since none of the prediction software predicted them to be deleterious. Finally, the screening of *ZNF480* (NM144684; 4 coding exons) a zinc finger protein which is located at 19q13.41, led to the identification of two nonsense variants in one SCZ patient and one control individual (R276X and R500X, respectively). During our gene screening process, five nonsense variations (R304X, R360X, R416X, K434X and Q528X) were deposited in the EVS database by other groups with frequencies ranging from 1/10 755 to 2/10 756. Although we cannot rule out that schizophrenic or borderline personalities can be found in public databases such as EVS, the fact that five additional nonsense variations were found in the C-terminal region of this gene is not in favor of a deleterious effect of a *de novo* nonsense mutation in this region. For this reason we did not further investigate this gene in additional SCZ cases and control individuals and concluded that *ZNF480* was a poor candidate for SCZ.

**Table 1:** Mutations identified in LRP1, ALS2CL, ZNF480 and KPNA1 and occurrence in SCZ and CTR cohorts

**Mutation details**									**Prediction scores**			**Occurrence**	
Gene	Genomic Position^a^	Nucleotide variant^b^	AA Change	Type^d^	dbSNP^e^	ESV^f^	1000 genomes^g^	Inheritance^h^	Panther^i^	Sift^j^	PolyPhen^k^	SCZ ^l^ cohort	CTR cohort
**LRP1**	**chr12**:**57**,**579**,**450**		**Y2200X**^**c**^	**NS**				***de novo***					
LRP1	chr12:57,538,812	c.506G > A	G169D	MS	-	A = 2 / G = 10756	-	N/A	-	0.01	2.337	1/189	-
LRP1	chr12:57,539,265	c.833G > T	S278I	MS	-	-	-	T (mother)	−2.47064	0.08	1.73	1/189	-
LRP1	chr12:57,548,392	c.1135G > A	R379H	MS	-	-	-	N/A	−2.23136	0.07	1.571	1/189	-
LRP1	chr12:57,574,263	c.5386 + 1G > A	M1795I	MS	-	-	-	T (mother)	-	0.06	1.968	1/189	-
LRP1	chr12:57,577,915	c.5977C > T	R1993W	MS	rs141826184	T = 25 / C = 10733	T = 1 / C = 1093	N/A	-	0.05	0.037	2/189	-
LRP1	chr12:57,578,673	c.6238G > A	D2080N	MS	rs34577247	A = 159 / G = 10599	A = 26 / G = 2162	N/A	-	0.45	0.375	>5	-
LRP1	chr12:57,579,328	c.6478G > A	A2160T	MS	-	A = 1 / G = 10495	-	N/A	-	0.60	1.147	1/189	-
LRP1	chr12:57,587,040	c.7637G > A	G2546S	MS	rs113379328	A = 24 / G = 10734	-	N/A	-	0.13	0.836	>5	-
LRP1	chr12:57,587,717	c.7840G > A	R2613Q	MS	rs150340911	A = 12 / G = 10746	-	N/A	-	0.36	0.898	2/189	-
LRP1	chr12:57,588,275	c.8057G > A	R2686H	MS	rs148104493	A = 1 / G = 10755	-	N/A	-	0.12	0.999	1/189	-
LRP1	chr12:57,589,784	c.8699A > C	Q2900P	MS	rs7397167	A = 123 / C = 10635	A = 14 / C = 2174	N/A	-	0.53	-	>5	-
LRP1	chr12:57,590,916	c.9044G > A	G3015S	MS	rs145303173	A = 6 / G = 10752	-	N/A	-	0.76	0.357	2/189	-
LRP1	chr12:57,598,513	c.11175G > A	G3725E	MS	rs151301245	A = 12 / G = 10746	-	T (mother)	-	0.03	1.583	1/189	-
LRP1	chr12:57,600,508	c.11843G > A	G3948D	MS	-	-	-	N/A	-	0.50	1.357	1/189	-
**ALS2CL**	**chr3**:**46**,**717**,**166**		**R733X**^**c**^	**NS**				***de novo***					
ALS2CL	chr3:46,717,175	c.2188C > T	G730S	MS	rs142971127	T = 80 / C = 10678	T = 12 / C = 2176	N/A	-	0.40	1.483	7/475	1/189
ALS2CL	chr3:46,718,458	c.1812G > T	P605T	MS	-	-	-	N/A	−1.70789	0.42	1.761	0/475	1/189
ALS2CL	chr3:46,718,477	c.1793C > T	R598H	MS	-	-	-	N/A	−2.73328	0.01	1.686	0/475	1/189
ALS2CL	chr3:46,719,769	c.1737G > A	P580S	MS	-	-	-	T (mother)	-	0.62	1.615	1/475	0/189
ALS2CL	chr3:46,719,861	c.1645T > C	N549S	MS	rs140347863	C = 9 / T = 10749	-	T (father). N/A. T (mother). N/A	-	0.35	1.851	3/475	1/189
ALS2CL	chr3:46,722,792	c.1380G > A	T460M	MS	-	A = 1 / G = 10757	-	T (father)	−2.00878	0.11	0.374	1/475	0/189
ALS2CL	chr3:46,725,290	c.894G > A	A298V	MS	rs141781567	A = 15 / G = 10757	-	N/A	−1.6125	0.14	1.366	2/475	0/189
ALS2CL	chr3:46,725,522	c.802T > A	T268S	MS	-	-	-	T (mother)	−1.48254	0.33	1.406	1/475	0/189
ALS2CL	chr3:46,728,477	c.530A > G	I176T	MS	rs145807890	G = 8 / A = 10746	-	N/A	−1.28647	0.39	1.351	0/475	1/189
ALS2CL	chr3:46,729,700	c.190C > A	E65X	NS	rs139496961	A = 20 / C = 10738	-	N/A. T (father). N/A. N/A	-	-	-	4/475	1/189
ALS2CL	chr3:46,729,756	c.134C > G	E45Q	MS	rs7642448	G = 4590 / C = 6166	-	N/A	-	0.28	1.042	>5	>5
**ZNF480**	**chr19**:**52**,**826**,**001**		**R500X**^**c**^	**NS**				***de novo***					
ZNF480	chr19:52,825,329	c.826C > T	R276X	NS	-	-	-	N/A	-	-	-	1/475	0/189
ZNF480	chr19:52,825,495	c.992C > A	A331E	MS	-	-	-	N/A	-	0.95	0.838	0/475	1/189
ZNF480	chr19:52,826,001	c.1498C > T	R500X	NS	-	-	-	N/A	-	-	-	0/475	1/189
**KPNA1**	**chr3**:**122**,**146**,**472**		**E448X**^**c**^	**NS**				***de novo***					
KPNA1	chr3:122,186,188	c.218C > T	S73N	MS	rs4678193	T = 79 / C = 10677	T = 11 / C = 2177	N/A	−2.16746	0.45	0.174	>5	-

^a^ according to build Hg19; ^b^ variant position according to Mutalyzer 2.0.beta-20 [12] and to Genbank accession number NM_002332.2 for *LRP1*, NM_147129.3 for *ALS2CL*, NM_144684.2 for *ZNF480* and NM_002264.3 for *KPNA1*; ^c^ MS: missense and NS: nonsense; ^d^ nonsense mutations previously identified by exome sequencing [6]; ^e^ rs number as obtained in dbSNP database [7]; ^f^ observed allele counts according to Exome Variant Server in all populations [9]; ^g^ observed allele counts according to 1000 genomes in all populations [8]; ^h^ inheritance study when parents available; ^i^ according to Panther, sub-PSEC score is the probability that a given coding variant will cause a deleterious functional change when less than −3 [13]; ^j^ according to Sift scores (from 0 to 1), the amino acid substitution is predicted to be damaging if the score is < = 0.05 and tolerated if the score is > 0.05 [11]; ^k^ according to polyphen, if PSIC score difference is > 1.5, the variation is predicted to be possibly or probably damaging [10,11]. ^l^ > 5 indicates that the variant has been identified in more than 5 individuals.

## 

While the results of this mutation screening effort did not lead to the identification of additional potentially deleterious *de novo* mutations in *LRP1*, *KPNA1*, *ALS2CL* and *ZNF480* in a larger cohort of SCZ patients, it was a necessary first step to assess their possible contribution to disease. One limitation of this study is that we have focused only on the coding regions. This does not exclude the involvement of disease predisposing variants in non-coding regions, which could affect allelic expression or splicing. Given the known heterogeneity of SCZ and the frequencies of variants reported thus far, the contribution of these particular genes may only emerge after the progressive sequencing of coding and non-coding regions of these genes in much larger cohorts of SCZ cases and control individuals. It’s likely that an additional mechanism (like the polygenic mode) is involved in the genetics of sporadic SCZ. However, we still believe that deleterious *de novo* mutations play an important role in a proportion of SCZ patients as demonstrated by our group and others [14-16]. It is well known that SCZ is genetically heterogeneous and hundreds and probably thousands of genes are involved. Therefore, to date, very few cases with *de novo* mutation have been reported to be associated with the same genes. Addressing the complete genetic picture of a polygenic disease such as SCZ is still a major challenge and will require further independent replication studies to clarify the role of these genes.

## Websites references

ExonPrimer: http://ihg.gsf.de/ihg/ExonPrimer.html.

Primer3 Plus interface: http://www.bioinformatics.nl/cgi-bin/primer3plus/primer3plus.cgi.

## Competing interests

The authors declare that they have no competing interests.

## Authors’ contributions

LJ, SLG, PAD and GAR designed the study. LJ, SD, AA performed the experiments. MOK and RJ recruited cases and collected clinical information. LJ, JG, PAD and GAR wrote the paper. All authors have approved the final manuscript.
